# Obesity and Prognostic Variables in Colombian Breast Cancer Patients: A Cross-Sectional Study

**DOI:** 10.1155/2017/9574874

**Published:** 2017-05-18

**Authors:** Javier Cuello-López, Ana Fidalgo-Zapata, Elsa Vásquez-Trespalacios

**Affiliations:** ^1^Clinical Oncology Group, Fundación Colombiana de Cancerología-Clínica Vida, Medellín, Colombia; ^2^Breast Surgeon Fellowship Program, School of Medicine, CES University, Medellín, Colombia; ^3^Department of Clinical Epidemiology, School of Medicine, CES University, Medellín, Colombia

## Abstract

**Introduction:**

Obesity is an established risk factor for cancer and cancer-related deaths, including that of the breast. While the prevalence of female obesity has accelerated over the past decade in many developing countries, such as Colombia, the prevalence of overweight and obesity specifically in breast cancer populations has not been fully described.

**Methods:**

A cross-sectional study including 849 women diagnosed with breast cancer between 2009 and 2014. Based on body mass index, prevalence of overweight (BMI ≥ 25 < 30) and obesity (BMI ≥ 30) and associations of BMI with clinical and tumor histopathological features were analyzed.

**Results:**

Colombian breast cancer patients had a prevalence of overweight of 34.28% and obesity of 28.15%. Mean BMI was comparable between premenopausal and postmenopausal women (27.2 versus 27.7, resp.). Among premenopausal women, higher BMI was significantly positively associated with hormone receptor negative tumors, as well as with greater lymphovascular invasion.

**Conclusions:**

Colombian breast cancer patients exhibit a significant prevalence of overweight and obesity. Associations of high BMI and poor prognosis variables in the premenopausal population suggest risk of aggressive disease in this population. Future studies to further validate our observations are warranted in order to implement multidisciplinary clinical guidelines.

## 1. Introduction

Overweight and obesity are current escalating public health issues. Close to one billion people worldwide are considered overweight (range of 25 to 29.0 kg/m^2^), and as many as 475 million are obese (BMI ≥ 30 kg/m^2^) [1]. Moreover, it is estimated that, by the year 2030, half of the world's population will be obese [2]. Moreover, in developing countries, obesity is an established risk factor for several cancer and cancer-related deaths [3].

Breast cancer is the leading cause of cancer and the second cause of cancer-related death worldwide [4]. It is estimated that globally, every year 1.671.149 new cases are diagnosed, and 521,907 patients succumb to this disease [5]. While incidence of breast cancer has steadily increased over the past decades, mortality rates are decreasing likely due to early detection and more effective therapies [6, 7]. Incidence rates vary nearly fourfold across the world regions, in less developed regions (324,000 deaths). In Colombia the annual incidence is 33,5 per 100,000 persons according to data Globocan 2012, with an annual mortality of 9,8 and estimated prevalence of 18.582 cases per 100,000 persons [5].

Furthermore, the identification of risk factors and comorbidities affecting breast cancer incidence or progression has become a matter of intense research [8].

The mechanisms by which obesity affects breast cancer incidence and progression are not fully understood; however, previous studies have proposed both direct and indirect mechanisms. Among the direct mechanisms are insulin resistance, inflammation, and altered adipokine profile (increased levels of leptin and decreased levels of adiponectin). These mechanisms can stimulate breast cells to increase estrogen production, which in turn will stimulate proliferation and signaling through the estrogen receptor (ER). Among the indirect mechanisms are included disease detection at advanced stage and subtherapeutic or increased treatment doses [1, 9–12].

Additionally, descriptive studies, systematic reviews, and meta-analysis have shown that obese breast cancer patients have worse survival in both pre- and postmenopausal women. This was observed regardless of whether the BMI was assessed before, during, or after treatment. Furthermore, at the time of diagnosis, obese patients have greater tumor size, grade, and more advanced stage [1, 9, 11, 13–16].

In Colombia, in 2005, 49.6% of women and 39.9% of men between the ages of 18 and 64 years were either overweight or obese [17]. More recent data suggest that this population is expanding, as in 2010 over half (51.2%) of the population of that same age group was overweight to some extent, being 34.6% overweight (BMI ≥ 25 ≤ 30) and 16.5% obese (BMI ≥ 30). In those studies, excess of weight was more prevalent in women than in men (55,2% versus 45,6%), especially as obesity (20,1% versus 11,5%). At the national level, morbid obesity (BMI ≥ 40) was 0.9% of the adult population, also being more prevalent in women than in men [17–19]. Collectively, these studies show that excess of weight, both as obesity and morbid obesity, is more prevalent in Colombian women than in men.

To date, in Colombia there are no local or national studies reporting the prevalence of obesity specifically in breast cancer patients. In this work we describe the prevalence of obesity in breast cancer patients and analyze the relationship of BMI and prognostic factors in this population.

## 2. Materials and Methods

### 2.1. Study Design and Participants

This was a cross-sectional study of 849 women diagnosed with breast cancer between January 2009 and December 2014. Participants were recruited of the* Fundación Colombiana de Cancerología-Clínica Vida*, in Medellín, Colombia. The Ethics Committee at our institution approved this study. Patient data was collected from available electronic databases.

### 2.2. Variables

Participant's body weight, height, and body mass index [BMI = weight (kg)/height^2^ (cm^2^)] were collected at the time of diagnosis. Patients were classified following the Center for Disease control's (CDC) criteria: underweight (BMI < 18.5 kg/m^2^), normal (18.5 ≤ BMI < 25 kg/m^2^), overweight (25 ≤ BMI < 30 kg/m^2^), and obese (BMI ≥ 30 kg/m^2^) [20].

Menopausal status was defined by one of the following criteria: age ≥ 60 years old; history of bilateral oophorectomy; amenorrhea for more than 1 year (in the absence of chemotherapy, tamoxifen, or ovarian suppression); postmenopause levels of FSH, LH, or estradiol. Patients with bilateral breast cancer or inadequate disease staging were excluded from the study.

The following prognostic clinical factors were analyzed: stage (TNM by AJCC 2010), histologic grade, and lymphovascular invasion. Expressions of hormone receptors (estrogen and progesterone receptors), Ki67, and Her2 receptor were detected by immunohistochemistry (IHC). FISH studies were performed in samples in which Her2 immunohistochemistry was inconclusive or borderline.

Based on IHC results, tumors were classified as the following subtypes: luminal A (ER + and/or PR+, Ki67 < 14%, and Her2-negative), luminal B negative (ER + and/or PR+, Ki67 ≥ 14%, and Her2-negative), luminal B positive (ER + and/or PR+, Ki67 ≥ 14%, and Her2-positive), HER2-enriched (ER-negative, PR-negative, and HER2-positive), and triple negative (ER/PR and Her2 negative) [21].

### 2.3. Data and Statistical Analysis

Data were analyzed using SPSS® (Windows version) software. For all tests, a *p* value < 0.05 was considered statistically significant. Associations between body weight and other variables were examined using Chi-square and Fisher tests.

Quantitative variables are shown as averages and their respective measures of statistical dispersion. Qualitative variables were represented as percentages. Statistical significance of averages was examined by Student's *t*-test (for independent samples) or Mann–Whitney *U* test as needed.

## 3. Results

### 3.1. Patient Characteristics

A total of 849 participants diagnosed with breast cancer were recruited for this retrospective study. Mean age was 54 years, ranging from 26 to 91 years old. Of these, 496 (58.42%) were premenopausal and the remaining 353 (41.58%) were postmenopausal. Based on BMI, patients were classified into four groups: underweight, normal, overweight, and obese. In our sample, there was a prevalence of overweight of 34.28% and of obesity of 28.15% (Table 1).

### 3.2. BMI and Menopausal Status

Mean BMI was comparable between pre- and postmenopausal women, being 27.2% in both groups (*p* = 0.443) (Table 2). While premenopausal women showed a slightly lower prevalence of obesity than postmenopausal participants (26.7% versus 31.1%), this trend was not statistically significant (*p* = 0.482) (Table 2).

### 3.3. Correlations of BMI and Breast Cancer Histopathologic Features

**Figure 1:**
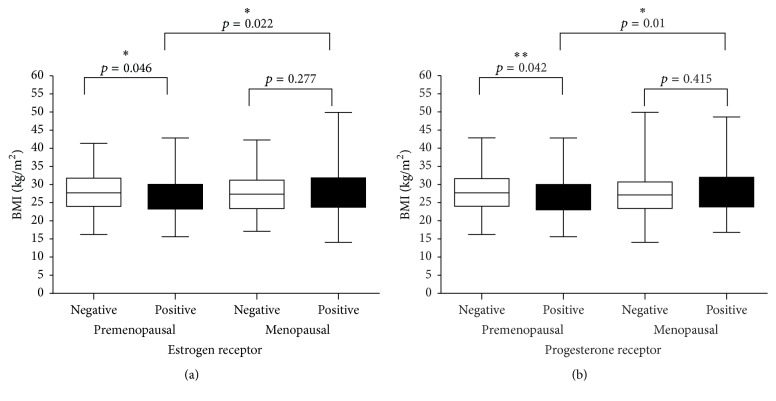
BMI and hormone receptor status in premenopausal and postmenopausal breast cancer patients. *∗* and *∗∗* refer to the level of significance.

Correlations between BMI and each of the tumor pathology variables were analyzed in pre- and postmenopausal groups. With regard to hormone receptor status, we found increased BMI to be associated with having ER-negative (*p* = 0.046) as well as PR-negative tumors (*p* = 0.042) in the premenopausal group. Furthermore, compared to normal-weight patients, BMI > 25 patients had a higher frequency of PR-negative tumors (30.3% versus 44.3%, *p* = 0.042) (Figure 1(a)). This correlation was not observed in the postmenopausal group (Figure 1(b)). Among obese patients, a correlation between high BMI and hormone receptor positive tumors was found in the postmenopausal group and not in the premenopausal group (Figure 1).

**Figure 2:**
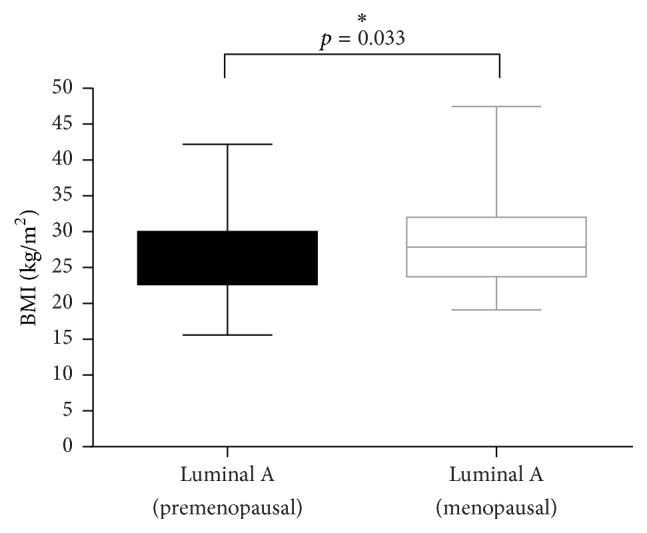
Higher BMI is associated with luminal A tumors in postmenopausal women. *∗* refers to the level of significance.

Regarding molecular subtypes, luminal A breast tumors were found to be associated with greater BMIs (*p* = 0.003) in the postmenopausal group. This correlation was not observed in the premenopausal group (Figure 2). There were no additional correlations found between BMI and the remaining molecular subtypes in either pre- or postmenopausal groups.

**Figure 3:**
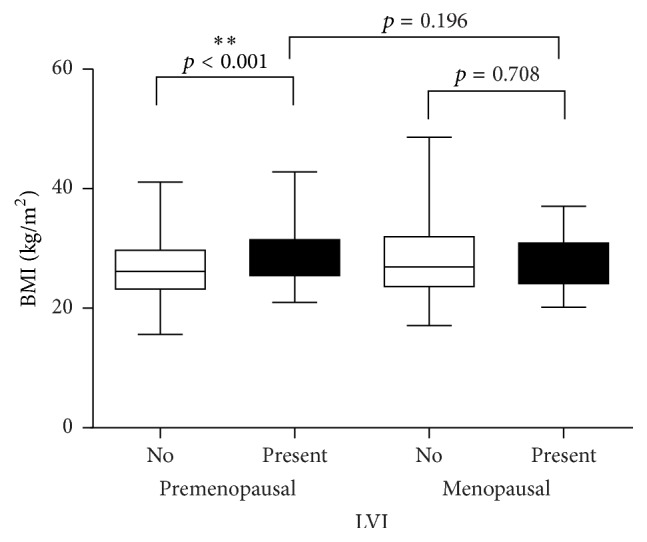
LVI is associated with higher BMI in premenopausal breast cancer patients. *∗∗* refers to the level of significance.

In premenopausal patients, increased BMI was associated with increased lymphovascular invasion (LVI) (Figure 3). Such a correlation was not found in the postmenopausal group.

Additional correlations between the BMI and the remaining tumor variables (tumor grade, lymph node involvement, and disease stage) were not significant in either pre- or postmenopausal women (Tables 3 and 4).

## 4. Discussion

In this study, we describe the prevalence of obesity in Colombian breast cancer patients and analyze the relationship of BMI with prognostic factors. The prevalence of obesity in our study population was 28.15%. While this prevalence is comparable to that reported for breast cancer patients in the United States [22], it is greater than the obesity prevalence described in European studies (13% to 20%) [16, 23, 24]. Collectively, these data suggest that there is a demographic factor in the prevalence of obesity in breast cancer patients [25].

Previous studies have shown overweight and obesity to be related to postmenopausal breast cancer. Several mechanisms have been proposed to explain this observation, including increased circulating estrogen levels and estrogen aromatization in adipose tissue. The latter decreases levels of sex hormone-binding globulin (SHBG), affecting aromatase gene expression regulation [23, 26]. However, unlike previous studies, an association between BMI and menopausal status was not statistically significant in our population. These results may be partly explained by the twofold increased prevalence of obesity in the premenopausal group in our study compared to that of previous reports (26.7% versus 10–14%) [22, 23].

Obese breast cancer patients are often associated with advanced stage disease, larger tumors, and axillary lymph node-positive status, partly due to delayed diagnoses and technical difficulties in palpation of the tumors [27–29]. However, in this study we did not find a statistically significant association between BMI and tumor size, lymph node-status, disease stage, or histological grade in the current study groups. It is plausible that a larger sample size may be required to establish this association.

In the context of lymphovascular invasion (LVI), Demirkan and colleagues showed that, in breast cancer patients with BMI ≥ 30 kg/m^2^, LVI was an independent prognostic factor of poor outcome [30]. In agreement with previous studies [31], our data show a strong association of LVI with BMI > 25 in the premenopausal group.

In this study, compared to premenopausal women, postmenopausal women with higher BMIs were found to be associated with luminal A tumors. This is consistent with previous reports describing a positive correlation between hormone receptor status and obese postmenopausal breast cancer patients in Europe [32]. On the other hand, a study by Millikan and colleagues reported an inverse relationship between BMI and hormone receptor status in premenopausal women [33], in agreement with our data. Additional studies suggest that tumors in obese breast cancer patients, particularly ER/PR-negative tumors, are dependent of growth factors such as insulin, insulin growth factor 1 (IGF1), and leptin [34].

This study is the first report, to our knowledge, of obesity prevalence in Colombian breast cancer patients. Our results indicate that there is a high prevalence of obesity in our study population. Because obesity is a recognized risk factor for poor outcome in breast cancer patients, especially in premenopausal females, Colombian women may be at higher risk of developing more aggressive disease. Future studies to further validate our observations are needed in order to implement multidisciplinary clinical care practices that may provide better care and targeted treatment for breast cancer patients.

## Figures and Tables

**Table 1:** Patient demographic and tumor histopathologic characteristics.

Characteristics	*N* = 849
Mean age at time of diagnosis in years (range)	54 (26–91)

Age groups, years, *n* (%)	
<35 years	41 (4.83)
35–39	41 (4.83)
40–49	245 (28.86)
50–59	223 (26.27)
60–69	172 (20.26)
≥70	127 (14.96)

Menopausal status, *n* (%)	*n* (%)
Premenopausal	496 (58.42)
Postmenopausal	353 (41.58)

BMI, mean (range), *n* (%)	27.4 (14–49.9)
Unknown	15 (1.7%)
<18.5	13 (1.53%)
18.5–24.9	291 (34.28%)
25–29	291 (34.28%)
≥30	239 (28.15%)

Tumor size, *n* (%)	*n* (%)
TX	25 (2.94)
T1	210 (24.73)
T2	301 (35.45)
T3	140 (16.49)
T4	173 (20.38)

Lymph node status, *n* (%)	
Nx	293 (34.51)
N0	304 (35.8)
N1–N3	252 (29.68)

TNM, *n* (%)	
I	176 (20.73)
II	389 (45.82)
III	228 (26.86)
IV	32 (3.77)

Histological subtype, *n* (%)	
Ductal invasive	711 (83.7)
Lobular invasive	57 (6.71)
Other	37 (4.36)

Tumor grade, *n* (%)	
G1	143 (16.84)
G2	375 (44.17)
G3	211 (24.85)

Estrogen receptor status, *n* (%)	
Positive	605 (71.26)
Negative	244 (28.74)

Progesterone receptor status, *n* (%)	
Positive	537 (63.25)
Negative	312 (36.75)

HER 2, *n* (%)	
Positive	211 (24.85)
Negative	601 (70.79)
Unknown	37 (4.36)

Tumor subtype (IHC4), *n* (%)	
Luminal A	298 (35.1)
Luminal B, HER 2−	172 (20.26)
Luminal B, HER 2+	131 (15.43)
Her 2-enriched	79 (9.31)
Triple negative	133 (15.67)

**Table 2:** BMI distribution.

Characteristics	Premenopausal	Postmenopausal	*p* value
	*N* = 496 (58.42%)	*N* = 353 (41.58%)	
BMI (kg/m^2^), range	27.2 (15.6–42.8)	27.7 (14–49.9)	0.443

Normal (18.5–24.9), *n* (%)	175 (35.7)	120 (34.9)	0.482
Overweight (25–29.9), *n* (%)	175 (35.7)	113 (32.8)	
Obese (≥30), *n* (%)	131 (26.7)	107 (31.1)	

**Table 3:** BMI and tumor histopathologic features in premenopausal Colombian breast cancer patients.

Tumor characteristics	BMI			
	Normal	Overweight	Obese	*p* value
Tumor size *n*, (%)				
TX	5 (2.9)	4 (2.3)	2 (1.5)	0.821
T1	45 (26.0)	39 (22.2)	26 (19.7)	
T2	60 (34.7)	59 (33.5)	52 (39.4)	
T3	31 (17.9)	39 (22.2)	23 (17.4)	
T4	32 (18.5)	35 (19.9)	29 (22.0)	

Lymph nodes *n*, (%)				
Nx	66 (13.7)	53 (11)	42 (8.7)	0.412
N0	57 (11.8)	70 (14.5)	55 (11.4)	
N1–N3	50 (10.4)	53 (11)	35 (7.2)	

TNM *n* (%)				
I	35 (7.2)	34 (7.0)	22 (4.5)	0.847
II	83 (17.26)	86 (17.8)	68 (14.1)	
III	42 (8.7)	49 (10.1)	37 (7.6)	
IV	8 (1.66)	4 (0.8)	3 (0.6)	

Tumor grade				
G1	36 (20.6)	30 (17.1)	17 (13.0)	0.142
G2	69 (39.4)	83 (47.4)	61 (46.6)	
G3	41 (23.5)	45 (25.7)	40 (30.5)	

Estrogen receptor status				
Positive	132 (75.4)	121 (69.1)	83 (63.4)	0.072
Negative	43 (24.6)	54 (30.9)	48 (36.6)	

Progesterone receptor status				
Positive	122 (69.7)	111 (63.4)	73 (55.7)	0.042
Negative	53 (30.3)	64 (36.6)	58 (44.3)	

HER2 status, *n* (%)				
Negative	123 (71.1)	115 (65.3)	95 (72.0)	0.384
Positive	42 (24.3)	54 (30.7)	35 (26.5)	
Unknown	8 (4.6)	7(4.0)	2 (1.5)	

Molecular subtype (IHC4)				
Luminal A	61 (34.9)	57 (32.6)	43 (32.8)	0.397
Luminal B, HER 2−	38 (21.8)	32 (18.3)	21 (16.0)	
Luminal B, HER 2+	30 (17.1)	33 (18.9)	19 (14.5)	
Her2-enriched	13 (7.4)	20 (11.4)	16 (12.2)	
Triple negative	25 (14.3)	26 (14.9)	30 (22.9)	

**Table 4:** BMI and tumor histopathologic features in postmenopausal Colombian breast cancer patients.

Tumor characteristics	BMI			
	Normal	Overweight	Obese	*p* value
Lymph nodes *n*, (%)				
Nx	36 (10.5)	45 (13.2)	44 (12.9)	0.068
N0	45 (13.2)	29 (8.5)	39 (11.4)	
N1–N3	37 (10.8)	41 (12)	24 (22)	

TNM *n* (%)				
I	26 (7.6)	27 (7.9)	28 (8.2)	0.879
II	51 (15.0)	48 (14.1)	45 (13.2)	
III	33 (9.7)	34 (10)	26 (7.6)	
IV	6 (1.7)	2 (0.5)	5 (1.47)	

Tumor grade				
G1	23 (19.2)	16 (14.2)	17 (15.9)	0.900
G2	52 (43.3)	49 (43.4)	51 (47.7)	
G3	29 (24.2)	28 (24.8)	24 (22.4)	

Estrogen receptor status				
Positive	85 (70.8)	86 (76.1)	80 (74.8)	0.635
Negative	35 (29.2)	27 (23.9)	27 (25.2)	

Progesterone receptor status				
Positive	70 (58.3)	73 (64.6)	71 (66.4)	0.415
Negative	50 (41.7)	40 (35.4)	36 (33.6)	

HER2 status, *n* (%)				
Negative	83 (70.3)	86 (74.8)	83 (77.6)	0.632
Positive	29 (24.6)	24 (20.9)	22 (20.6)	
Unknown	6 (5.1)	5 (4.3)	2 (1.9)	

Molecular subtype (IHC4)				
Luminal A	46 (38.3)	34 (30.1)	46 (43.0)	0.287
Luminal B, HER 2−	20 (16.7)	36 (31.9)	21 (19.6)	
Luminal B, HER 2+	19 (15.8)	15 (13.3)	13 (12.1)	
Her2-enriched	11 (9.2)	7 (6.2)	9 (8.4)	
Triple negative	18 (15.0)	17 (15.0)	16 (15.0)	
